# Comparison of EASYDO ACTIVATOR, passive ultrasonic, and needle irrigation techniques on the treatment of apical periodontitis: a study in rats

**DOI:** 10.1007/s00784-022-04677-6

**Published:** 2022-09-02

**Authors:** Qin Ye, Yao Feng, Ya-Qiong Zhao, Li Tan, Jing Hu, Shao-Hui Zhang, Jie Zhao, Ying-Hui Zhou, Yue Guo, Yun-Zhi Feng

**Affiliations:** 1 Department of Stomatology, The Second Xiangya Hospital of Central South University, Changsha, Hunan, 410011 China; 2 National Clinical Research Center for Metabolic Diseases, Hunan Provincial Key Laboratory of Metabolic Bone Diseases, and Department of Metabolism and Endocrinology, The Second Xiangya Hospital of Central South University, Changsha, Hunan 410011 China

**Keywords:** Apical periodontitis, EASYDO ACTIVATOR, Passive ultrasonic irrigation, Needle irrigation, TNF-α, IL-6

## Abstract

**Objectives:**

To evaluate the long-term therapeutic effect of EASYDO ACTIVATOR, passive ultrasonic irrigation, and needle irrigation in experimental apical periodontitis in rats.

**Materials and methods:**

Sprague-Dawley male rats were used to produce periapical lesions. The pulp chambers of the bilaterally first mandibular molars were exposed and left open for 21 days. The rats were divided into four groups according to different irrigation protocols. Seven days after irrigation, the mandibles were removed for micro-CT, histological, and immunohistochemical analysis. Serum levels of tumor necrosis factor-alpha (TNF-α) and interleukin-6 (IL-6) were assessed by enzyme-linked immunosorbent assays (ELISA). Statistical data were analyzed by one-way analysis of variance (ANOVA) with LSD tests.

**Results:**

The passive ultrasonic irrigation and EASYDO ACTIVATOR groups had the smallest apical lesions compared to the other groups (*P* < 0.05), while the needle irrigation group had smaller lesions than the control group (*P* < 0.05). The EASYDO ACTIVATOR group had less inflammation infiltration compared to the control and needle irrigation groups (*P* < 0.05). The control and needle irrigation groups had more TNF-α expression compared to the passive ultrasonic irrigation and EASYDO ACTIVATOR groups (*P* < 0.05). The lowest IL-6 expression was observed in the EASYDO ACTIVATOR group. The EASYDO ACTIVATOR group had the lowest serum level of TNF-α than other groups (*P* < 0.05). IL-6 expression was significantly lower in the EASYDO ACTIVATOR group in comparison with the control and needle irrigation groups (*P* < 0.05).

**Conclusions:**

EASYDO ACTIVATOR can significantly reduce the apical lesions and decrease the inflammatory response around the periapical area.

**Clinical relevance:**

EASYDO ACTIVATOR is recommended for clinical application.

## Introduction

Apical periodontitis is an inflammatory disease in the periapical tissue caused by bacterial infections in the root canal system [1, 2], which consequently lead to inflammation in the apical area and alveolar bone resorption [3]. Bone resorption has been regarded as a part of the host defense process, and multiple inflammatory mediators play a role in this process, including tumor necrosis factor-alpha (TNF-α) and interleukin-6 (IL-6) [4]. The success of treatment depends on eliminating remaining microorganisms or colonization in the root canal, which is hard to achieve because of the complexity of the root canal system [5]. Studies have shown that mechanical preparation is able to reach only the central body of the root canal [6], leaving the remaining irregularities untouched and biofilms as a possible source of treatment failure [7]. Therefore, irrigation of the root canal systems is considered an essential part of disinfection of the unprepared root canal [8].

Needle irrigation is the most commonly used irrigation method. However, with this method, the apex does not receive enough irrigation because the irrigants can reach only approximately 1 mm beyond the needle tip [9]. Thus, different irrigation activation systems have been proposed to improve the cleanliness and disinfection of the root canal system [10, 11]. Passive ultrasonic irrigation is an irrigation system with non-cutting files to activate the irrigants in the root canal by ultrasonic frequencies [12]. Studies have shown that compared with syringe irrigation, ultrasonic irrigation can better remove microorganisms and the smear layers on root canal walls [12, 13]. However, as the depth of the root canals increased, the effectiveness of passive ultrasonic irrigation for canal cleanliness and disinfection seemed to decrease [14]. According to the manufacturer, the EASYDO ACTIVATIOR system uses the technology of vibrational energy for root canal treatment. The system helps the fluids to stream into the pulp chamber, root canal, lateral canal, and connection of inter-root canal and root apex to ensure deep cleaning and disinfection of the root canal using a highly flexible tip, which can memory which mode you used.

Multiple studies have been carried out to evaluate the efficacy of different irrigation systems using ex vivo extracted teeth or artificial grooves [15–18]. It has been proved that different irrigation devices can help remove the debris and smear layers and improve sealer penetration, but their influence on the inflammation process during apical periodontitis needs further investigation [15]. A study in pigs evaluated the effect of irrigation systems on the removal of biofilms [19]. However, the apical periodontitis model in pigs is very expensive, which may limit its application. Researchers have used rats as experimental apical periodontitis models and assessed the changes in the lesions by different measures, such as hematoxylin and eosin (H&E) staining and micro-CT analysis [20, 21]. In these models, root canal treatment (RCT) significantly reduced the bacterial count in the root canal, establishing a system leading to clinical healing. Thus, with this model, it is possible to evaluate the changes and reactions occurring as a result of irrigation before RCT.

The aim of this study is to determine whether different irrigant activation techniques improve therapeutic effects at animal level and provide support for later clinical application. The null hypothesis tested is that different irrigation techniques do not have any effects on apical periodontitis.

## Materials and methods

**Fig. 1:**
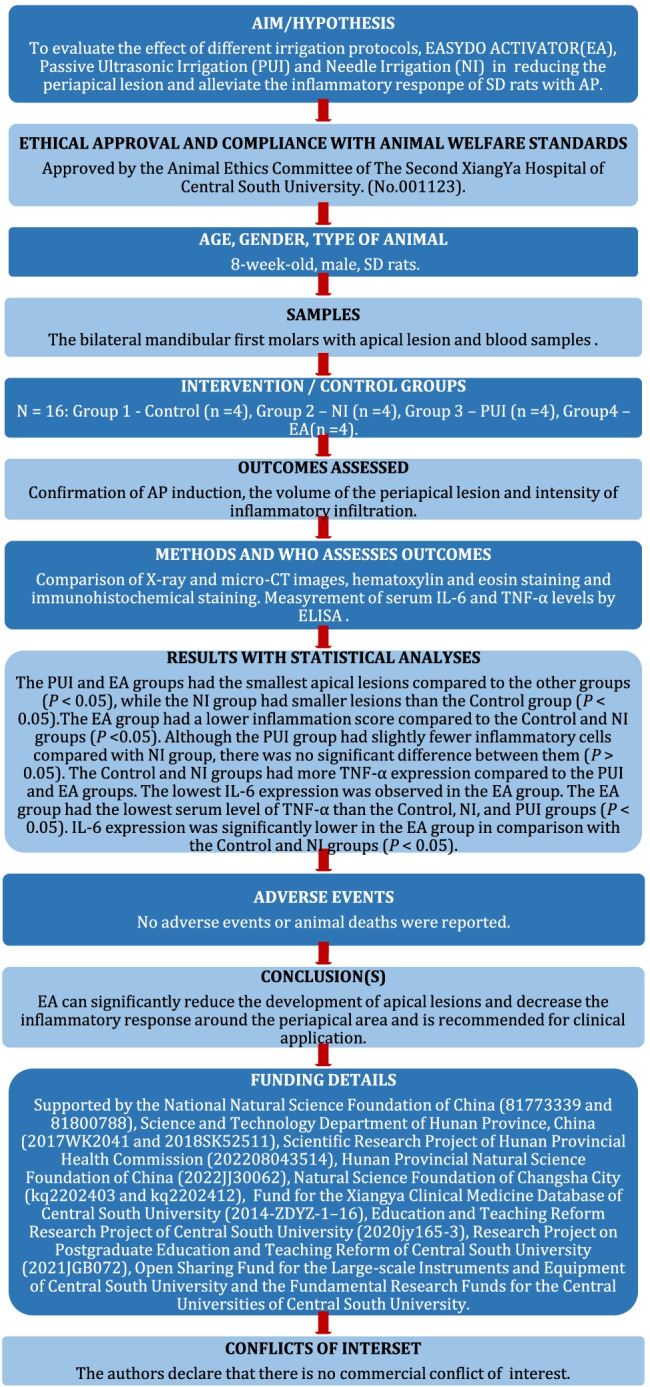
PRIASE 2021 flowchart illustrating the steps involved in conducting the present study

The manuscript of this animal study has been written according to Preferred Reporting Items for Animal studies in Endodontology (PRIASE) 2021 [22] guidelines (Fig. 1).

### Animals

The experimental protocol of this study was approved by The Animal Ethics Committee of The Second XiangYa Hospital of Central South University (No. 001123). The rats were housed in a specific-pathogen-free (SPF) animal unit under standardized conditions: temperature (23 ± 2 °C), 12 h light/dark cycle (lights on at 07:00), and relative humidity (55 ± 10%) and fed a pelleted diet ad libitum.

### Experimental design

Sixteen 8-week-old male Sprague-Dawley rats (200-250 g) were divided into four experiment groups. The sample size was calculated based on the study of a similar design [21]. The experimental design is as follow:

Firstly, the apical periodontitis model was induced as previously described [21]. The rats were anesthetized with an intraperitoneal injection of 3% sodium pentobarbital solution. The pulp chamber of the bilateral mandibular first molar of each rat was exposed by a 1/4 round dental burr, at a speed of 800 revolutions per minute (rpm). Then the pulp was taken out by a barbed broach (#000, MANI, Japan) and the pulp cavity was left open to the oral environment for 21 days to allow periapical lesion formation. The above surgery was performed under a stereomicroscope (Phenix, XTL-168, China). Soft food was provided because the rats were incapable of eating hard food because of tooth pain. After 21 days, one rat was randomly selected from each group and their radiographs of the bilateral mandibular alveolar bone were taken to confirm that the apical periodontitis models were successfully established.

After the periapical lesion induction period, the remaining twelve rats were anesthetized and the root canals were prepared up to a #10 K-file (Dentsply, Ballaigues, Switzerland) at the depth of 2.5 mm into the mesial root canal. Then the remaining twelve rats were randomly divided into four experimental groups, according to different irrigation procedures: group 1 (control), no irrigation was done; group 2 (needle irrigation), irrigation was done with 1.5 ml 3% NaOCl using a 30-G side-vented needle at a flow rate of 1 mL/min; group 3 (passive ultrasonic irrigation), same as group 2 but with passive ultrasonic activation of the irrigants using a VDW ULTRA system (VDW, Munich, Germany); group 4 (EASYDO ACTIVATOR), irrigants were activated by the EASYDO ACTIVATOR (Easyinsmile, Changsha, China) with 1.5 ml 3% NaOCl.

Then, all the canals were dried with absorbent paper points (VDW, Munich, Germany) and the corona filled with Cavit G (3 M ESPE, MN, USA) for 7 days. Left mandibular molars were taken to radiologic analysis, while the other side of mandibular alveolar was send to histological and immunohistochemical analysis.

### X-ray imaging

After dissection, the mandible specimens were fixed in 4% paraformaldehyde phosphate buffer solution for 48 h. The mandible specimens were put in a culture dish (10 mm diameter), and the X-ray photographs were taken from the buccolingual direction at a setting of 35 kV, 2 mA for 4 min to observe the periapical lesions.

### Micro-CT analysis

The rat mandible specimens were dissected and fixed in 4% paraformaldehyde phosphate buffer solution for 48 h and then were preserved in 70% alcohol until use. Periapical lesions were scanned by a micro-CT machine (μCT50, Scanco, Bassersdorf, Switzerland) at settings of 90 kV and 160 µA. After scanning, the images were reconstructed by the VGSTUDIO MAX30 (v3.0, Volume Graphics, Germany). The region of interest (ROI) was measured in the low-density space around the mesial root of first mandibular molars as the volume of the apical lesion.

### Histological analysis

The specimens were fixed in 4% paraformaldehyde phosphate buffer solution and decalcified in 17% EDTA for 21 days. After dehydration with serial concentrations of ethanol (70-100%), the samples were embedded in paraffin. Serial Sects. (4 µm) were cut in a mesiodistal orientation. The sections exhibiting a root canal apex and the periapical tissues of first molars were selected for H&E staining and observed under a light microscope (Leica Microsystems, Wetzlar, Germany). The periapical lesion size (in mm^2^) in the periapical region was measured and the severity of periapical lesion was scored as previously described [23, 24]:

- Score 1 (absent): 0 to few inflammatory cells;
- Score 2 (mild): < 25 inflammatory cells; inflammation limited to the apex;
- Score 3 (moderate): 25-125 inflammatory cells; inflammation present in the apical third of the root or in the middle third;
- Score 4 (intense): > 125 inflammatory cells; widely diffuse inflammation, possibly in the furcation defect.

### Immunohistochemical staining

After deparaffinization and rehydration, the 4-µm sections were treated with antigen retrieval solution, 0.25% trypsin, at 37 ℃, 30 min and then incubated with 3% H_2_O_2_ for 30 min. The samples were blocked with 1% bovine serum albumin (Servicebio, Wuhan, China) in phosphate buffered saline (PBS, 1 × Servicebio, Wuhan, China) for 30 min at room temperature and were incubated with the following primary antibodies overnight at 4 °C: TNF-α (Abcam, ab220210; Species: Rat, Human; Host: Mouse; England) and IL-6 (Novusbio, NB600-1131; Species: Human, Mouse, Rat, Monkey; Host: Rabbit; USA). Slides then were washed three times in PBS and incubated with a biotinylated secondary antibody (Servicebio, Wuhan, China) for 1 h at room temperature. After being rinsed in PBS, the slides were incubated with the avidin-biotin-peroxidase complex for 20 min. Twenty microliters of 50 × DAB stock solution was added to each 1 mL of DAB dilution solution for later use as a chromogen. The slides were counterstained with hematoxylin and rinsed under running water. Image analysis was performed by one skilled evaluator through an optical microscope (Leica Microsystems, Wetzlar, Germany).

### Enzyme-linked immunosorbent assay

After the rats were euthanized, the whole blood samples were collected in a centrifuge tube at room temperature for 2 h and then centrifuged (1000 × *g*, 15 min) and the serum was collected and stored at - 80 °C. Serum levels of TNF-α and IL-6 were measured by enzyme-linked immunosorbent assay (ELISA) kits (Cusabio, Wuhan, China). The procedures were carried out according to the instructions provided with the kits. The reactions were done in triplicate. Optical density (OD) value of each hole was measured sequentially at 450 nm wavelength. The regression equation of the standard curve was calculated according to the concentration and OD value of the standard and the standard curve was constructed. The OD value of the sample was substituted into the equation to calculate the sample concentration.

### Statistical analysis

All values were expressed as means ± standard deviation (SD). Statistical analysis was performed using SPSS software (SPSS Statistics, v23.0; SPSS Inc., IBM, Armonk, NY, USA). Statistical analysis was performed by one-way analysis of variance (ANOVA) with LSD tests between multiple groups. All *P* values less than 0.05 were considered significant.

## Results

### Radiologic analysis

**Fig. 2:**
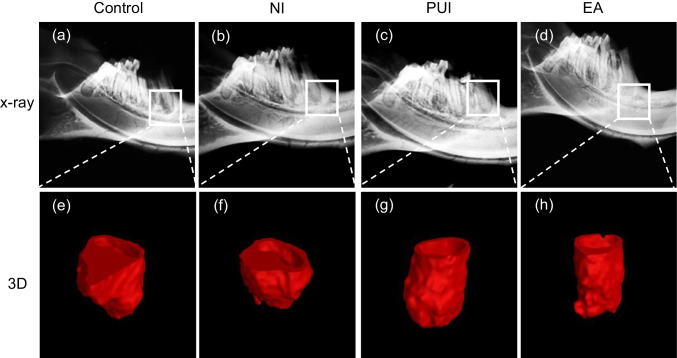
Representative radiologic image of the rats apical lesion of at 7 days after different irrigation procedures. X-ray images of the mandibular lesion of the control group (**a**), needle irrigation group (**b**), the passive ultrasonic irrigation group (**c**), and the EASYDO ACTIVATOR group (**d**). Representative 3D reconstruction of the apical lesion of the control group (**e**), the needle irrigation group (**f**), the passive ultrasonic irrigation group (**g**), and the EASYDO ACTIVATOR group (**h**). Control, the control group; NI, the needle irrigation group; PUI, the passive ultrasonic irrigation group; EA, the EASYDO ACTIVATOR group

Figure 2 shows the X-ray images of the mandibular lesion and representative 3D reconstruction of the apical lesion. The largest lesions were observed in the control group and the smallest lesions in the passive ultrasonic irrigation and EASYDO ACTIVATOR groups.

**Fig. 3:**
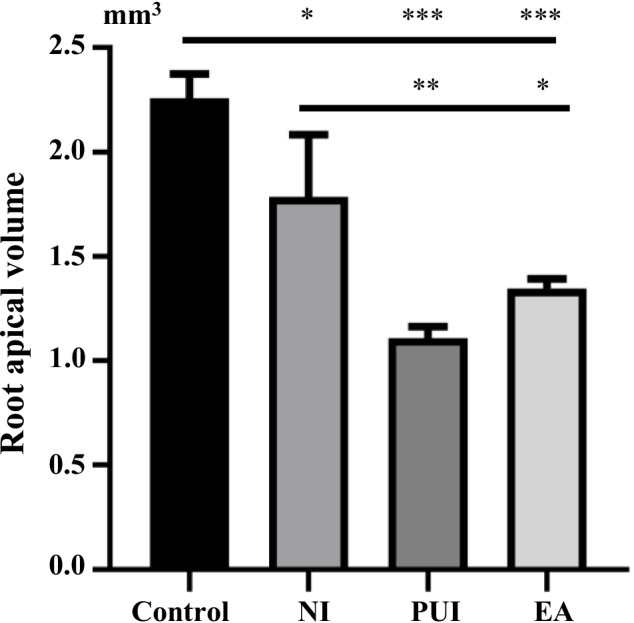
The quantitative analysis of root apical volume in different groups. The control group had larger apical lesions than the needle irrigation group and then the passive ultrasonic irrigation and EASYDO ACTIVATOR groups. Control, the control group; NI, the needle irrigation group; PUI, the passive ultrasonic irrigation group; EA, the EASYDO ACTIVATOR group.* < 0.05, ** < 0.01, *** < 0.001

The quantitative analysis (Fig. 3) showed that the lesion volume in the control group was significantly larger than in the other groups (*P* < 0.05). The needle irrigation groups had larger apical lesions than the passive ultrasonic irrigation and EASYDO ACTIVATOR groups (*P* < 0.05). There was no significant difference in the lesion volume of passive ultrasonic irrigation and EASYDO ACTIVATOR groups (*P* > 0.05).

### Histological analysis

**Fig. 4:**
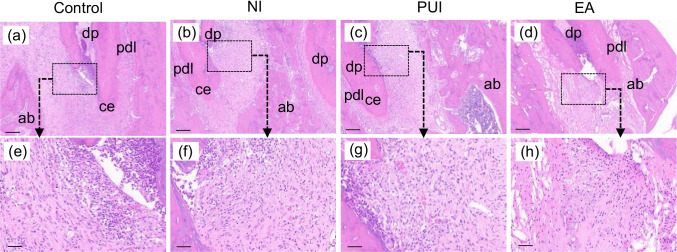
Representative histological images of periapical tissues at 7 days after different irrigation procedures. **a** and **e** Control group - severe inflammatory infiltration was observed in the apical region. **b** and **f** Needle irrigation group - similar degree of inflammatory infiltration was observed as the control group. **c** and **g** Passive ultrasonic irrigation group - decreased inflammatory infiltration in the apical region compared to the control group. **d** and **h** EASYDO ACTIVATOR group - the degree of inflammatory infiltration was significantly reduced. Scale bar = 200 μm. (**e**)-(**h**) are higher magnification of the images shown in (**a**)-(**d**). Control, the control group; NI, the needle irrigation group; PUI, the passive ultrasonic irrigation group; EA, the EASYDO ACTIVATOR group. dp, dental pulp; pld, periodontal ligament; ce, cementum; ab, alveolar bone. Scale bar = 50 μm

Histological analysis was performed to evaluate the influence of different irrigation devices on periapical inflammation (Fig. 4). In the control group, there were multiple inflammatory cells, mainly neutrophils and mononuclear cells, infiltrating in the periapical area. The apical lesion was widely extended. According to quantitative analysis of the inflammatory infiltration intensity (Table 1), the control group had the severest inflammation compared to the passive ultrasonic irrigation and EASYDO ACTIVATOR groups (*P* < 0.05). The EASYDO ACTIVATOR group had a significant reduction in the periapical inflammation infiltrate compared to the needle irrigation group (*P* < 0.05). Although the passive ultrasonic irrigation group had slightly fewer inflammatory cells compared with the needle irrigation group, there was no significant difference between them (*P* > 0.05).

**Table 1:** The mean score (means ± SD) of the intensity of inflammatory infiltration in different groups

Inflammation Score	Groups			
	Control	NI	PUI	EA
1-Absent	0/6	0/6	0/6	0/6
2-Mild	0/6	0/6	3/6	4/6
3-Moderate	3/6	5/6	2/6	2/6
4-Severe	3/6	1/6	1/6	0/6
Mean ± SD	3.5 ± 0.55^a^	3.17 ± 0.41^ac^	2.67 ± 0.82^bc^	2.33 ± 0.52^b^

*NI*, needle irrigation; *PUI*, passive ultrasonic irrigation; *EA*, EASYDO ACTIVATOR. *Different letters indicate significant statistical differences in rows (*P* < 0.05)

### Immunohistochemical analysis

**Fig. 5:**
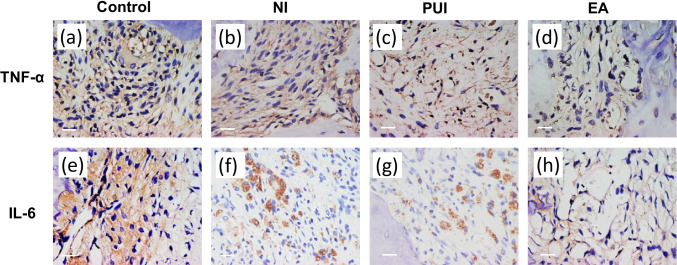
Immunohistochemical staining for TNF-α and IL-6 in the periapical tissues 7 days after different irrigation procedures. TNF-α expression in the periapical tissues of the control group (**a**), the needle irrigation group (**b**), the passive ultrasonic irrigation group (**c**), and the EASYDO ACTIVATOR group (**d**). In situ IL-6 expression in the periapical tissues of the control group (**e**), the needle irrigation group (**f**), the passive ultrasonic irrigation group (**g**), and the EASYDO ACTIVATOR group (**h**). Control, the control group; NI, the needle irrigation group; PUI, the passive ultrasonic irrigation group; EA, the EASYDO ACTIVATOR group. Scale bar = 20 μm

The expression of TNF-α and IL-6 in the periapical area is shown in Fig. 5. TNF-α and IL-6 were found in the periapical lesions, mostly clustered around the apical foramen. TNF-α was also localized in alveolar bone lacunae and root cementum. The control and needle irrigation groups had greater TNF-α expression compared to the passive ultrasonic irrigation and EASYDO ACTIVATOR groups. IL-6 was mainly expressed in the extracellular region. The control group had greater IL-6 expression compared to the needle irrigation and passive ultrasonic irrigation groups. The lowest IL-6 expression was observed in the EASYDO ACTIVATOR group.

### Serum levels of TNF-α, IL-6

Serum levels of TNF-α and IL-6 are shown in Table 2. The EASYDO ACTIVATOR group showed the lowest expression level of TNF-α compared to the control, needle irrigation, and passive ultrasonic irrigation groups (*P* < 0.05). There was no difference in the level of TNF-α between the needle irrigation group and the control group. The passive ultrasonic irrigation group had a lower level of TNF-α compared to the control but was not significantly different than that of the needle irrigation group.

**Table 2:** Serum level (mean ± SD) of TNF-α and IL-6 in different groups

Inflammatory mediators (pg/mL)	Groups			
	Control	NI	PUI	EA
TNF-α	6.34 ± 0.99^a^ 21.49 ± 2.32^a^	6.08 ± 0.56^ab^ 21.44 ± 3.56^a^	5.45 ± 0.34^b^ 19.82 ± 4.43^ab^	4.56 ± 0.41^c^ 17.28 ± 3.11^b^
IL-6				

*NI*, needle irrigation; *PUI*, passive ultrasonic irrigation; *EA*, EASYDO ACTIVATOR; *TNF-α*, tumor necrosis factor alpha; and *IL-6*, interleukin-6 in pg/mL. *Different letters indicate significant statistical differences in rows (*P* < 0.05)

IL-6 expression was significantly lower in the EASYDO ACTIVATOR group in comparison with the control and NI groups (*P* < 0.05). Rats in the EASYDO ACTIVATOR group showed a slight decrease in the serum level of IL-6 compared with rats in the passive ultrasonic irrigation group, but there was no significant difference between them (*P* > 0.05). No statistical differences in the IL-6 level were observed in the control, needle irrigation, and passive ultrasonic irrigation groups (*P* > 0.05).

## Discussion

Cleaning and shaping procedures are the key factors in controlling inflammation in the root canal and periapical areas [25]. Many new endodontic instruments have been introduced and used extensively in endodontics to deliver irrigants into the area after instrumental preparation, which has been shown to be an effective way to reduce apical inflammation [11, 26]. The aim of this study was to evaluate the efficacy of root canal treatment with different endodontic irrigation techniques in vivo using the rat model.

At 1 week after irrigation, we took root apex X-ray and micro-CT images of rats for study of the changes in apical bone, and the results showed that the volume of periapical lesions in the control group tended to increase in size compared to the other groups after 3 weeks of the pulp exposure, which is consistent with studies of radiographic measurements using Wistar rats [27, 28]. Furthermore, we targeted only the mesial root of the mandibular first molar in our study and measurement of the periapical lesion volume for micro-CT because the mesial root alone reflects a more accurate treatment efficacy [21]. Applying passive ultrasonic irrigation and EASYDO ACTIVATOR instruments could significantly alleviate the apical periodontitis compared to control and needle irrigation treatments. Tanaka et al. proved that the application of passive ultrasound could facilitate biofilm removal in the pig model [19]. Munoz et al. also compared endodontic irrigation systems for irrigant delivery in clinic and showed that irrigant delivery systems were more effective than conventional endodontic needles in delivering irrigant to root canals [29]. Furthermore, we undertook histological analysis to evaluate the progression of periapical lesion healing by observing the inflammatory infiltrate and new cementum formation [23]. We observed that the EASYDO ACTIVATOR and passive ultrasonic irrigation treatments had fewer inflammatory cells infiltrating the periapical area. These results were consistent with the micro-CT analysis, which further verified that application of the EASYDO ACTIVATOR and passive ultrasonic irrigation instruments could reduce inflammation residue.

Previous studies have proved that new irrigation systems could remove infected dentin and decrease root canal and apical inflammation in the root canal more effectively by delivering irrigants as compared with needle irrigation [11, 30]. It has been found that passive ultrasonic irrigation could effectively use acoustic streaming and cavitation and is more effective than syringe irrigation in removing pulp tissue remnants, dentin debris, microorganisms, and the smear layer, while EASYDO ACTIVATOR has been claimed to create a three-dimensional movement to trigger “cavitation” and “acoustic streaming” - two physical effects which up to now have only been seen with passive ultrasonic irrigation [15, 24]. Furthermore, the EASYDO ACTIVATOR instrument is a cordless handpiece that activates highly flexible polyamide, which may be more suitable for a small area and anatomical complexity of the in vivo operation, which may improve the efficiency of cleaning root canals [9]. Thus, it is reasonable to consider that the EASYDO ACTIVATOR and passive ultrasonic irrigation have a positive impact on cleaning root canals and reduce the development of periapical inflammation infiltrate.

TNF-α and IL-6 are the commonly found bone resorption-related factors and inflammatory cytokines during periodontitis. TNF-α stimulates the production of collagenase, prostaglandin E2, chemo- and cytokines, cellular adhesion molecules, and bone resorption-related factors [31, 32]. IL-6 acts as a proinflammatory cytokine during periodontitis and stimulates osteoclastic differentiation and bone resorption in chronic inflammatory periodontitis [33]. TNF-α and IL-6 were found in the periapical lesions, mostly clustered around the apical foramen. Littlle wood-Evans et al. found that the accumulation of TNF-α in the bone microenvironment was associated with the histopathological changes observed during bone resorption in rat models [34]. de Oliveira et al. found that reducing IL-6 immunoexpression in gingiva of rat molars decreased bone loss through reduction of osteoclasts by inhibiting MMP-1 and MMP-9 expression in the inflamed gingival mucosa [35]. Another study showed that upregulation of genes pertinent to inflammation (i.e., TNF-α, IL-6) could promote angiogenesis (i.e., VEGF, VWF) and osteogenesis (ALP, RUNX2, BGLAP, SP7) for bone regeneration and exhibits efficient osteoformation capacity [36]. Combined with the histological results and immunohistochemical staining of TNF-α and IL-6 in the periapical area, we proved that the passive ultrasonic irrigation and EASYDO ACTIVATOR treatments could reduce the levels of TNF-α and IL-6, thereby reducing bone destruction in apical periodontitis.

Oral inflammation could elevate systemic cytokine levels and cause pathological changes in remote organs [37]. Cotti et al. demonstrated that the presence of apical periodontitis was correlated with higher blood levels of IL-6 [38, 39]. Recent studies also revealed that patients with apical periodontitis face a higher risk of developing coronary artery disease [38, 40]. Our results showed that the EASYDO ACTIVATOR group had the best effect in reducing serum inflammatory factor, but the concentrations of TNF-α or IL-6 in the blood were not significantly different between the passive ultrasonic irrigation and the needle irrigation treatments. This indicated that there may be a relationship between the levels of serum inflammatory factors and the treatment effect of root canal irrigation, and the groups with better treatment effect had lower levels of serum inflammatory factors.

Under the experimental conditions of this study, although periapical bone resorption was observed, the healing process increased after root canal preparation and irrigation, and we believe that longer observation and root canal filling or antimicrobial therapy might be needed to draw conclusions about irrigation with different techniques [7, 41]. Also, root canal treatment increased healing speed and could be undertaken to better understand the healing process [21], so we suggest EASYDO ACTIVATOR or passive ultrasonic irrigation as an adjuvant treatment for apical periodontitis.

## Conclusions

Effective root irrigations, such as passive ultrasonic irrigation and EASYDO ACTIVATOR, can significantly reduce the size of apical lesionsand decrease the inflammatory response around the periapical area, which provide new technical direction for clinical treatments.
